# The safety and effectiveness of ultrasound-guided instrumental removal of retained placenta after failed removal immediately after delivery

**DOI:** 10.1515/crpm-2024-0051

**Published:** 2025-11-27

**Authors:** Zhiyin Wang, Yan Zhou, Lu Wang, Xuecui Xu, Yali Hu, Yimin Dai

**Affiliations:** Center for Obstetrics and Gynecology, Department of Obstetrics and Gynecology, 66506 Nanjing Drum Tower Hospital, Affiliated Hospital of Medical School, Nanjing University, Nanjing, P.R. China

**Keywords:** retained placenta, ultrasound-guided instrumental removal, placenta accrete

## Abstract

**Objectives:**

To evaluate the safety and effectiveness of ultrasound-guided instrumental removal of retained placenta.

**Case presentation:**

We presented a retrospective single-center cohort study include 54 patients diagnosed with retained placenta after delivery. The characteristics of the patients with retained placenta, who received ultrasound-guided instrumental removal, were evaluated. We also compared the characteristics between the patients with retained placenta with and without suspected placenta accrete.

**Conclusion:**

Curettage should be performed in retained placenta without suspected placenta accrete. In retained placenta with suspected placenta accrete, we could also perform ultrasound-guided instrumental removal therapy if the retained placenta was not in the lower uterine segment or in previous scars.

## Introduction

Retained placenta is an important contributor for postpartum hemorrhage, which is associated with maternal morbidity and mortality [1], [2], [3]. The incidence of retained placenta varied from 0.5 to 3 % [4], 5]. The main risk factors for retained placenta include preterm birth, congenital uterine anomaly, multiple pregnancy and maternal age [5], [6], [7]. Usually, retained placenta has been managed by manual placenta removal, which increased the risks of infections and postpartum endometritis [7], [8], [9]. In 2014, Rosenstein et al. proposed that ultrasound-guided instrumental removal of the retained placenta might reduce the discomfort to the patients [10].

The heterogeneity in retained placenta consists of three main types: placenta adherens (a normal placenta, insufficient contraction of the retroplacental myometrium), trapped placenta (a normal placenta, due to a contracted cervix or a structural uterine abnormality) and partial accrete (an abnormal adherent placenta [accrete]) [6], 11]. The pathophysiology of retained placenta is also complex, including invasive placentation, placental hypoperfusion and inadequate myometrial contractility [5].

The standard treatment for retained placenta is manual extraction. Due to that the instrument entering the uterus is much narrower than a hand, instrumental removal causes less discomfort to the patient than a traditional manual extraction. Curettage may lead to both immediate perioperative complications (including hemorrhage, infection, uterine perforation, and anesthesia-related risks) and long-term sequelae (encompassing intrauterine adhesions [Asherman’s syndrome], endometrial injury [e.g., this endometrium], and secondary infertility). Current studies remain insufficient regarding postoperative surveillance for women with partially retained placenta undergoing ultrasound-guided instrumental removal.

Due to the challenges in distinguishing subtypes of partially retained placenta, establishing a clinical consensus on treatment approaches remains problematic [12]. Some postpartum women are still diagnosed with partially placental retention or placenta accrete by ultrasound examination, who failed to intrapartum treatment such as manual removal of placenta or conservative treatment. Universally, placenta accrete is a contraindication for ultrasound-guided instrumental removal. However, the management of patients only based on ultrasound examinations are not rigorous. In our unit, we actively perform ultrasound-guided instrumental removal for these postpartum women with partially retained placenta. However, ultrasound-guided instrumental removal of retained placentas carries risks for the patients with suspected partial placenta accrete. The upper and middle segments of the uterus have stronger contractility than the lower segment. Thus, we speculated that we could perform a curettage in patients with suspected placenta accrete if the location is not in the lower uterine segment or previous scars. This retrospective study aimed to assess the safety and effectiveness of ultrasound-guided instrumental removal for the patients with retained placenta.

## Subjects and methods

### Study design

It is a single-center retrospective case series study performed in Nanjing Drum Tower Hospital, a referral tertiary care center. Permission was obtained from our Institutional Ethics Committee before the study (2024-205-01). A total of 108 women with retained placenta after midtrimester delivery between January 2018 and December 2023 were reviewed. The inclusion criteria were:1) retained placenta after delivery; 2) stable hemodynamic state; 3) no antenatal suspicion of placenta accrete; 4) failed manual removal of the placenta immediately after vaginal delivery. The exclusion criteria were: 1) placenta overlying previous cesarean section scar; 2) pseudoaneurysm of the uterine artery, arterio-venous malformations; 3) successfully instrumental removed placenta after delivery; 4) conservative therapy; 4) laparotomy and no attempted instrumental removed the placenta; 5) incomplete data. The diagnosis of placenta retention was made when placenta partial expulsion or incomplete removal after delivery, and a well-defined tissue mass within the uterine cavity was found by ultrasound scan.

### Basic demographics and characteristics of study population

**Figure 1:**
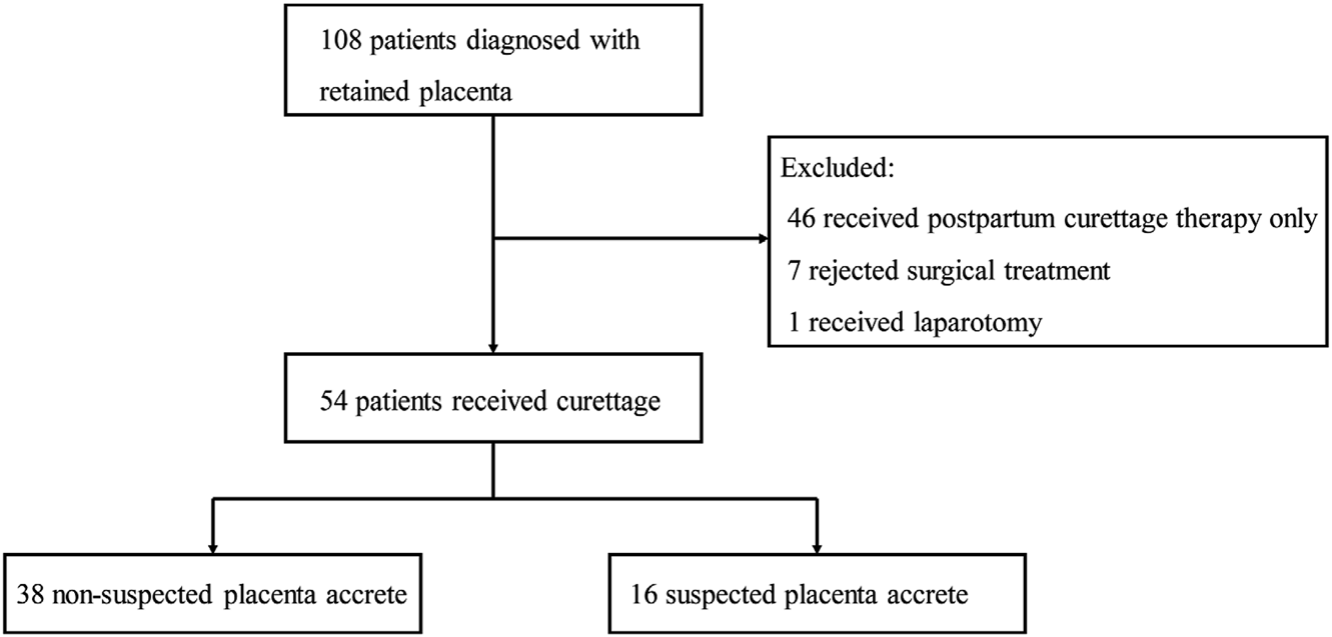
Flow chart of excluded patients for the analysis of women with retained patients who were performed by curettage.

From January 2018 and December 2023, a total of 108 patients, who delivered in our unit or were referred from external hospitals, were included. 46 patients, who received postpartum curettage as only therapy, were excluded. 62 patients were diagnosed with partially retained placenta by ultrasound examination. Eventually, 54 patients were included in our study with the exclusion of seven rejected surgical treatments and one performed laparotomy. These patients failed in conservative management and received an ultrasound-guided instrumental removal of retained placentas (Figure 1). Detailed demographic information was presented in Table 1. The majority of patients were nullipara (57.5 %) between 22 and 43 years of age (median 31.0 years, IQR 27.8–33.0 years). A total of 50.0 % women underwent previous uterine cavity surgery and 20.4 % had previous cesarean section. Vaginal delivery (85.2 %) played a dominant role in all patients and the gestational age ≥37 weeks accounted for 55.6 % women.

**Table 1:** Basic characteristics and ultrasound characteristics of all women with retained placenta.

	All (n=54)
**Maternal basic characteristics**	
Age, years	31.0 (27.8–33.0)
Nullipara	31 (57.5 %)
Gravidity	2.0 (1.0–3.0)
History of uterine surgery	27 (50.0 %)
History of cesarean section	11 (20.4 %)
Vaginal delivery	46 (85.2 %)
Cesarean delivery	8 (14.8 %)
Gestational age at delivery	
≥37 weeks	30 (55.6 %)
<37 weeks	24 (44.4 %)
Singleton pregnancy	48 (88.9 %)
Twin pregnancy	6 (11.1 %)
IVF in this pregnancy	3 (5.6 %)
**Intrapartum and postpartum basic characteristics**	
Manual removal at delivery	21 (38.9 %)
Curettage of uterine cavity at delivery	14 (25.9 %)
PPH (≥500 mL)	26 (48.1 %)
PPH (≥1,000 mL)	11 (20.4 %)
Postpartum mifepristone therapy	22 (40.7 %)
Postpartum methotrexate therapy	9 (16.7 %)
Postpartum DSA	2 (3.7 %)
Postpartum sepsis	2 (3.7 %)
**Ultrasound characteristics**	
Tissue volume, cm^3^)	129.4 (38.2–488.4)
Location	
Fundus	27 (49.1 %)
Upper and middle part	10 (18.2 %)
Lower part	7 (13.0 %)
Uterine cervix	5 (9.3 %)
Unclear	8 (14.5 %)
Myometrial vascularity	
Minimal	13 (23.6 %)
High	41 (75.9 %)
Suspected partial placenta accreta	16 (29.6 %)

Data are median (interquartile range) or n (%). IVF, *in vitro* fertilization; PPH, postpartum hemorrhage; DSA, digital subtraction angiography.

Among 54 patients, 38.9 % women also received manual removal of retained placenta after delivery. As shown in Table 1, the proportion of postpartum hemorrhage (PPH) ≥500 and 1,000 mL were 48.1 % and 20.4 %, respectively. Among these patients, 40.7 % women received mifepristone therapy, 16.7 % systematic methotrexate and 3.7 % UAE therapy after delivery. A total of two patients experimented sepsis in these all 54 patients. All patients had evidence of ultrasound diagnosis before admission. The median tissue volume of retained placenta in patients was 129.4 cm^3^ (IQR 38.2–488.4 cm^3^). Meanwhile, 29.6 % were suspected placenta accrete by ultrasound examination.

### Clinical management

During the study period, active management of retained placenta were as follows. Women with retained placenta were all offered planned surgical removal under ultrasound guidance, except placenta overlying previous cesarean section scar before delivery. These women often experienced postpartum hemorrhage, several attempts of evacuation, and even endometritis or infections caused by prolonged residual placentas. Thus, anemia needed to be corrected and intravenous administration of broad-spectrum antibiotics required before surgery. Detailed ultrasound examination was performed by experienced operators via both transvaginal and transabdominal. The location in the uterine cavity and the volume of the placenta mass, thinning of the myometrium overlying the placenta were recorded. The suspected placenta accrete were reported based on the guideline from Federation International of Gynecology and Obstetrics (FIGO) [13]. Briefly, loss of the retroplacental hypoechoic boundary, increased vascularity between the uterine muscle layer and the posterior wall of the bladder were prompted for placenta increta. The treatment was left to the discretion of treating clinicians. The procedure was performed in dorsal lithotomy under intravenous anesthesia by experienced obstetricians depending on their clinical symptoms and personal preferences.

### Treatment success

The successful ultrasound-guided instrumental removal of retained placentas defined as no massive hemorrhage (intraoperative hemorrhage <1,000 mL), no conversion to laparotomy or uterine artery embolization (UAE) during the operation, and no further surgical procedure needed. Severe postoperative complications in this work included conversion to laparotomy during curettage procedure, intraoperative hemorrhage ≥1,000 mL, uterine artery embolization (UAE), admission to intensive care unit (ICU) and new-onset sepsis.

### Variables

The following variables were included in this study: maternal age, nullipara, gravidity, previous uterine surgery, previous cesarean section, mode of delivery, gestational age, fetus number, *in vitro* fertilization, manual removal at delivery, curettage of uterine cavity at delivery, postpartum therapy, postpartum UAE, postpartum sepsis, ultrasound characteristics, blood indicators of preoperation and postoperation, type of anesthesia, operative duration, time between delivery and curettage, conversion to laparotomy, intraoperative hemorrhage, blood transfusion, admission to ICU, new-onset sepsis, postoperative UAE, postoperative hospital time, pathological inflammation of retained placenta and culture in any body fluid.

### Statistical analysis

Statistical analyses were carried out using SPSS 22.0 (SPSS, Chicago, IL, USA). Continuous data were presented as median and interquartile range (IQR). Categorical variables were tested by Fisher’s exact test and continuous variables were tested by Mann-Whitney *U* test. We used Spearman’s rank correlation analysis to investigate the correlation of tissue volume of retained placenta and operative duration, intraoperative hemorrhage or postoperative hospital time. A p-Value of <0.05 was deemed significant throughout.

## Results

### The outcome of patients with retained placenta received ultrasound-guided instrumental removal

A total of 54 women with retained placenta, who decided to receive ultrasound-guided instrumental removal. In almost all cases (98.1 %) ultrasound-guided instrumental removal has been performed successfully, in one case (1.9 %) a laparotomy was necessary (Table 2). The median of intraoperative hemorrhage was 50.0 mL (IQR 20.0–200.0 mL) and three (7.3 %) experimented intraoperative hemorrhage ≥1,000 mL, one treated with intensive care unit (UAE) after surgery. Only one patient was admitted to ICU and no women had new-onset sepsis. Overall, there were only four patients (7.4 %) with severe postoperative complications. The detailed characteristics of each patients were showed in Table 3.

**Table 2:** Postoperative characteristics of all women with retained placenta.

Characteristics of curettage treatment	All (n=54)
General anesthesia	54(100 %)
Operative duration, min	20.0 (12.8–30.0)
Time between delivery and curettage, days	26.0 (12.5–51.0)
Conversion to laparotomy	1 (1.9 %)
Intraoperative hemorrhage, mL	50.0 (20.0–200.0)
Intraoperative hemorrhage ≥500 mL	5 (9.3 %)
Intraoperative hemorrhage ≥1,000 mL	3 (5.6 %)
Blood transfusion	8 (14.8 %)
Admission to ICU	1 (1.9 %)
New-onset sepsis	0
Postoperative DSA	1 (1.9 %)
Postoperative hospital time, days	3.0 (2.0–5.3)
Pathological inflammation of retained placenta	11 (20.4 %)
Culture positive in any body fluid	8 (14.8 %)

Data are median (interquartile range) or n (%). ICU, intensive care unit; DSA, digital subtraction angiography; WBC, white blood cell; GR, granulocyte; LY, lymphocyte; ANC, absolute neutrophil count; LF, lymphocyte; PLT, platelet; HGB, hemoglobin; HCT, hematocrit value; NLR, international normalized ratio; PLR, platelet-to-lymphocyte ratio. ΔHGB (g/l): difference in hemoglobin levels preoperation minus postoperation. ΔHCT (%): difference in hematocrit value preoperation minus postoperation.

**Table 3:** The detailed characteristics of each patient with severe postoperative complications.

Patient number	1	2	3	4
Maternal basic characteristics				
Age, years	25	31	27	24
Nullipara	No	No	Yes	Yes
Gravidity	3	4	3	1
Parity	0	2	1	1
History of uterine surgery	2	2	1	0
History of cesarean section	0	2	1	0
Vaginal delivery	Yes	Yes	Yes	Yes
Singleton/twin pregnancy	Singleton	Singleton	Singleton	Singleton
IVF in this pregnancy	No	No	No	No
Intrapartum and postpartum basic characteristics				
Manual removal at delivery	Yes	No	Yes	Yes
Curettage of uterine cavity at delivery	No	Yes	No	Yes
Gestational age at delivery ≥37 weeks	Yes	No	No	Yes
PPH (≥500 mL)	Yes	Yes	Yes	Yes
PPH (≥1,000 mL)	No	No	No	Yes
Postpartum mifepristone therapy	Yes	Yes	Yes	Yes
Postpartum methotrexate therapy	Yes	No	Yes	Yes
Postpartum DSA	Yes	No	Yes	Yes
Sepsis of postpartum	No	No	No	Yes
Ultrasound characteristics				
Tissue volume, cm^3^	581.581	652.935	379.2	385
Location	Upper and middle	Upper and middle	Upper and middle	Upper
Myometrial vascularity	Minimal	Minimal	High	High
Suspected partial placenta accreta	No	Yes	Yes	No
Characteristics of curettage treatment				
Operative duration, min	35	10	120	19
Time between delivery and surgery, days	60	51	95	26
Conversion to laparotomy	No	Yes	No	No
Intraoperative bleeding volume, mL	1,000	1,200	1,600	50
Intraoperative hemorrhage ≥1,000 mL	Yes	Yes	Yes	No
Postoperation blood transfusion	Yes	Yes	Yes	No
Admission to ICU	No	No	No	Yes
Postoperative DSA	Yes	No	No	No
Postoperative hospital time, days	8	17	7	14
Pathological inflammation of retained placenta	No	Yes	Yes	No
Culture positive in any body fluid	No	Yes	No	Yes

Data are median (interquartile range) or n (%); IVF, *in vitro* fertilization; PPH, postpartum hemorrhage; DSA, digital subtraction angiography.

### Evaluation of the ultrasound in patients with partially retained placenta

The ultrasound confirmed the existence of partially retained placenta. In our 54 patients with retained placenta, there were 16 patients whose preoperative ultrasound suspected partial placenta accrete (Table 4). We found that the time between delivery and surgery in the suspected partially placenta accrete group was shorter than that in the non-suspected group (8 [4–14] vs. 33.5 [2.3–8.8] days, p=0.005). In addition, postoperative hospital time in the suspected partially placenta accrete group was longer than that in the non-suspected group (5.5 [2.3–8.8] vs. 2 [1–4] days, p=0.003). We also found that there was no residual placenta located in the lower segment of the uterus in the suspected group. There was no difference in severe postoperative complications between non-suspected and suspected partial placenta accrete.

**Table 4:** Patients with retained placenta diagnosed of non-suspected and suspected placenta accrete.

Characteristics of curettage treatment	Partial placenta accreta		p-Value
	Non-suspected	Suspected	
	n=38	n=16	
Operative duration, min	21.5 (15.0–30.0)	18.5 (10–25.0)	0.194
Time between delivery and surgery, days	33.5 (17.8–57.0)	8 (4–14)	**0.005**
Conversion to laparotomy	0 (0 %)	1 (6.3 %)	0.296
Intraoperative hemorrhage, mL	50 (20–200)	100 (50–187.5)	0.427
Intraoperative hemorrhage ≥500 mL	4 (10.5 %)	1 (6.3 %)	1.000
Intraoperative hemorrhage ≥1000 mL	2 (5.3 %)	1 (6.3 %)	1.000
Blood transfusion	6 (15.8 %)	2 (12.5 %)	1.000
Admission to ICU	1 (2.6 %)	0 (0 %)	1.000
Postoperative DSA	1 (2.6 %)	0 (0 %)	1.000
Postoperative hospital time, days	2 (1–4)	5.5 (2.3–8.8)	**0.003**
Lower location of residual placenta	7 (18.4 %)	0 (0 %)	0.09
Pathological inflammation of retained placenta	6 (15.8 %)	5 (31.3 %)	0.359
Culture positive in any body fluid	3 (7.9 %)	5 (31.3 %)	0.074

Data are median (interquartile range) or n (%), ICU, intensive care unit; DSA, digital subtraction angiography.

## Discussion

In our retrospective survey, the patients with partially retained placenta were all cured by ultrasound-guided instrumental removal therapy. Only four patients exhibited severe postoperative complications who have had all vaginal deliveries. The location of placental tissues of suspected placenta accrete group in our study were all not in the lower uterine segment or previous scars. In addition, there was no significant difference in severe postoperative complications between non-suspected and suspected placenta accrete group.

The retained placenta remains a difficult obstetrical problem worldwide [14], [15], [16]. Currently, manual removal is a standard management which could lead to major hemorrhage, endometritis, or partially retained placental tissue [7]. The retained portions of placentas could cause delayed hemorrhage or infection. With the help of medical treatment, partial placentas could be naturally excreted in some patients, while some patients still could not be managed through medical treatment. If the subtype of the retained placenta is partial accrete, a separation plane could not be created. However, current imaging examinations have limited accuracy between abnormally invasive and adherent placenta. In this situation, the ultrasound-guided instrumental removal is contraindicated, which could lead to massive hemorrhage [7], 17]. Some studies have described the expectant management which refers to the placenta diagnosed of partial accrete [18], [19], [20]. In fact, we treat the patients with partially retained placentas as a single entity and perform ultrasound-guided instrumental removal actively in our units. In this retrospective study, we aim to evaluate the effectiveness and safety of ultrasound-guided instrumental removal of partially retained placenta.

Currently, there is no standard management of retained placenta. The members of the European Working group on Abnormally Invasive Placenta (EW-AIP) have proposed a flow chart based on six cases to aid clinical management of retained placenta after vaginal delivery [12]. They preferred to manually remove the placenta when there is no excessive bleeding. If failed, they recommended to leave placentas *in situ* or perform hysterotomy with removal of placenta, local resection or hysterectomy. These six cases were all vaginal delivery and the size of the retained placenta was small. However, in this study, we included the patients with retained placentas after cesarean delivery. In our 54 patients, there were 38 patients with non-suspected placenta accrete and 16 patients with suspected placenta accrete. In patients with non-suspected placenta accrete, we performed ultrasound-guided instrumental removal therapy. If the suspected placenta accrete was not in the lower uterine segment or not in previous scars, we performed ultrasound-guided instrumental removal therapy actively although ultrasound examination suggests suspected placenta accrete. In our suspected placenta accrete group, there was no residual placenta located in the lower segment of the uterus. Thus, we performed ultrasound-guided instrumental removal of partially retained placenta actively. Eventually, only four patients exhibited severe postoperative complications. The postpartum hemorrhage, manual removal at delivery and sepsis of postpartum could cause poor basic physical condition, which may be related to severe postoperative complications.

**Figure 2:**
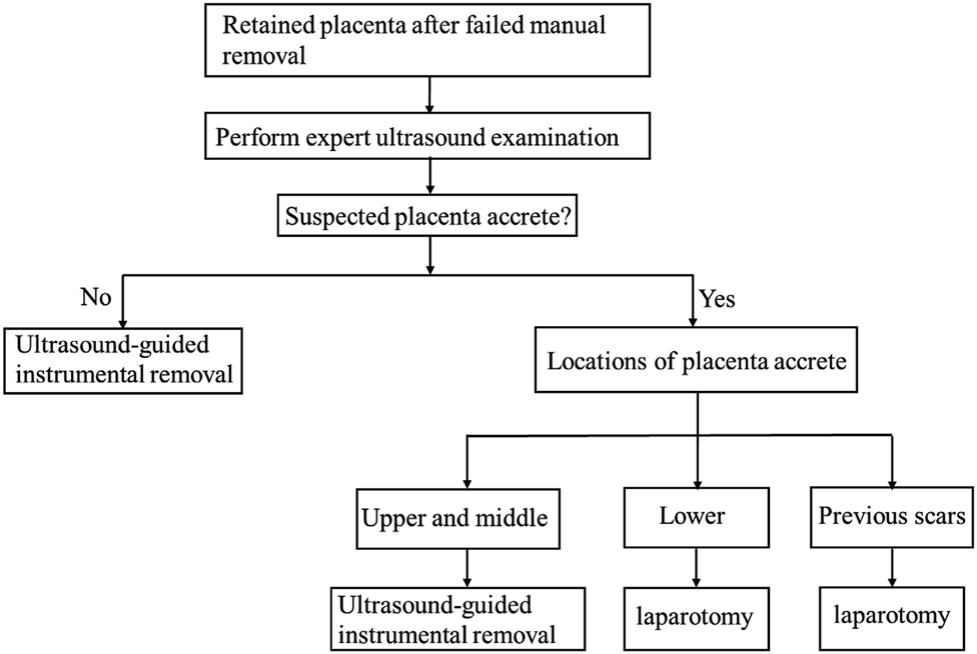
Flow chart for the management of retained placenta.

Our findings showed that we preferred not delay treatment timing due to suspicion of placenta accrete by ultrasound examination. Based on our results, curettage is relatively safe for patients with suspected placenta accrete if the location of placenta accrete is not in the lower uterine segment or in previous scars. Therefore, we designed a flow chart to aid consideration of management of retained placenta (Figure 2). The limitations of our research include, a small sample size and a retrospective design. Prospective studies and randomized controlled trials are needed to verify the effectiveness and safety of ultrasound-guided instrumental removal therapy in patients with retained placentas within the larger population.
